# Experimental Setup for Investigating the Efficient Load Balancing Algorithms on Virtual Cloud

**DOI:** 10.3390/s20247342

**Published:** 2020-12-21

**Authors:** Bhavya Alankar, Gaurav Sharma, Harleen Kaur, Raul Valverde, Victor Chang

**Affiliations:** 1 Department of Computer Science and Engineering, School of Engineering Sciences and Technology, Jamia Hamdard, New Delhi 110062, India; balankar@jamiahamdard.ac.in (B.A.); gaurav.0004@gmail.com (G.S.); 2 John Molson School of Business, Concordia University, Montreal, QC G1X 3X4, Canada; raul.valverde@concordia.ca; 3 Artificial Intelligence and Information Systems Research Group, School of Computing, Engineering and Digital Technologies, Teesside University, Middlesbrough TS1 3BX, UK

**Keywords:** cloud computing, load balancing/balancer, round robin, least connections, virtual machine, HAProxy, Apache Jmeter

## Abstract

Cloud computing has emerged as the primary choice for developers in developing applications that require high-performance computing. Virtualization technology has helped in the distribution of resources to multiple users. Increased use of cloud infrastructure has led to the challenge of developing a load balancing mechanism to provide optimized use of resources and better performance. Round robin and least connections load balancing algorithms have been developed to allocate user requests across a cluster of servers in the cloud in a time-bound manner. In this paper, we have applied the round robin and least connections approach of load balancing to HAProxy, virtual machine clusters and web servers. The experimental results are visualized and summarized using Apache Jmeter and a further comparative study of round robin and least connections is also depicted. Experimental setup and results show that the round robin algorithm performs better as compared to the least connections algorithm in all measuring parameters of load balancer in this paper.

## 1. Introduction

Technological innovations are surprising the human race every day. Cloud computing is one of them. It is emerging as a new umbrella field of various new applications as well as services. Rarely any field remains untouched by it. Its importance is significantly seen and felt today. There is no pre-defined or standard definition of cloud computing. Usually, it contains a cluster of servers known as controller servers that necessitate services and assets to different users in a network with scalability and reliability of the datacenter [1]. Virtualization technology allows us to work with powerful and multiple virtual machines, tweak-based configurations, and obtain the resources with core, RAM, and storage, to run on the same physical machine. The cloud provides 24/7 cost-free support, an open-source platform, automation tools, speed up the delivery, cloud-based applications, and much more. High the demand for cloud services and high-traffic web applications requires a highly capable well-organized load balancer for a smooth process of their industry. In a cloud environment, load balancing is applied on a cluster of servers and is used to achieve a variety of daemons, including Hypertext Transfer Protocol, Hypertext Transfer Protocol Secure, File Transfer Protocol, Simple Mail Transfer Protocol, Domain Name System, Post Office Protocol/Internet Message Access Protocol, and other requests [2].

Load balancing refers to proficiently allocating incoming network traffic to a cluster of backend servers. The primary key function of the load balancer is to make sure that each workstation of the network is utilized with an equally distributed amount of work. The load is not only for the web application traffic; it has various types like CPU load, network load, memory capacity concerns, etc. In the cloud computing environment, load balancing is used to allocate virtual machines across all end-user devices to improve asset utilization and provide high gratification to the audience [3]. This means neither of the workstations in the cloud goes overloaded or underutilized. In general, a load balancer acts as a reverse proxy that allocates network or web application traffic across a number of servers. Load balancers endorse three primary purposes. First, refining complete servers’ performance by accomplishing a high resource consumption ratio. Second, evading the server’s bottleneck that happens due to improper load balance. Third, it is important to attain high providers’ and audience’s gratification by increasing the servers’ throughput and decreasing the processing time load balancing assistances in preserving workstations’ insistence, performance, and security against workstation failures [4].

Recent high‑visitors’ net programs serve more than thousands of concurrent requests from audiences or clients and return the precise text, photographs, video, or application data, completely short and consistent. In order to correctly price the scale to satisfy these high potentials, contemporary computing great practices typically call for including extra servers. A load balancer acts as the “website visitors cop” sitting in the front of your servers and routing customer requests across all clusters of servers, proficient in accomplishing one’s requests in a way that will increase pace and capacity usage and confirm that no one server is overloaded (which could affect overall performance). If a single server is going down, the load balancer relays traffic to the active closing servers. Whilst a new server is delivered to the server cluster, the load balancer routinely starts to send requests to it.

In enormous datacenters, an additional quantity of load balancers is essential to balance the work across various web servers. Different load balancing algorithms like round robin, weighted round robin, and IP-based hashing are used in load balancers. All these algorithms do not see the live traffic before accelerating the request to the servers and promise not to run random algorithms on the load balancers as they are dealer specific and not run on product hardware and custom software [5]. The load balancer equally allocates the information subject to how demanding the server is. Without a load balancer, the audience would face delays in processing the information that might be annoying. At the time of load balancing progression, information like incoming tasks and vCPU (virtual CPU) processing rates are substituted among the vCPUs. Any downtime in the web application of load balancers can amount to severe penalties such as statistics loss. Several corporations use different types of load balancers, along with various load balancing algorithms. One of the most commonly used algorithms is the “round robin” or “least connections” load balancing.

This paper aims to investigate the role of using load balancer algorithms on live cloud infrastructure and open-source services, which leads to the reduction of the cost of the services. After experimental results, this paper also suggests that the round robin algorithm gives better performance results that help users choose the best algorithm for load balancing in their high web traffic applications.

This paper is organized as follows: Section 2 describe the background, types of load balancers, and its measurement parameters. Section 3 describes related works. Section 4 is on the methodology and experimental setup and practical implementation of the paper. Section 5 includes comparing two used load balancing algorithms and shows the results and evaluation of the test cases. The final Section 6 describes the conclusions of this paper.

## 2. Background

### 2.1. Types of Load Balancer

In the Introduction section, we discussed the load balancer. Our main aim is to give a brief idea of types of load balancers and how cloud users can choose the load balancer in cloud applications according to their requirements. There are some unique styles of load balancing that exist. We all know about network infrastructure, inclusive of Maria DB Server or SQL. Server load balancing for a relational database, worldwide server load balancing for troubleshooting across several geographic locations, and domain name server load balancing to assure domain name call functionality. We can also recall varieties of load balancers in phrases of the numerous cloud-based balancers: AWS Elastic Load Balancer, DigitalOcean Load Balancer, and so forth.

- Network Load Balancer: A network load balancer or Layer 4 Balancer uses the fourth layer of the OSI Model, which means it picks the information from the network layer for the route the network visitors that are dead through layer four load balancing and should handle the incoming request of TCP/UDP website visitors. Among a variety of load balancers, Network Load Balancer is the quickest load-balancer. Sometimes, it performs incline to drop once it routes the incoming network visitors across web application servers.
- HTTP(S) Load Balancer: The HTTP(S) Load balancer works on the application layer or seventh layer of the OSI model. HTTP uses session’s ids, cookies, and HTTP headers to decide how the network traffic or web visitors will be routed across all the web application clusters.
- Internal Load Balancer: Internal load balancing works on the layer 4 OSI model, similar to network load balancing. Internal load balancing is mostly implemented in onsite infrastructure to manage, stabilize, and balance the physical servers and virtual machines, including network area storage.
- Hardware Load Balancer: A hardware load balancer is a physical device that comes with a pre-installed operating system. Its role is to distribute or allocate the web users’ traffic across all the web application server farms (A server farm is a collection of webservers with network area storage on which web application hosted). Hardware Load Balancer requires a minimum of two virtual machines. It is configured by the system administrator with their custom rules to ensure the best performance, and the virtual machines are not overloaded. Hardware Load Balancer is not affordable for every user because it is very expensive, and it is dependent on the architecture and, hardware appliance of the infrastructure.
- Software Load Balancer: A software load balancer is a software-defined balancer that can be easily installed and configured on x86/64 bit servers or virtual machines. A minimum of four virtual machines are required for the software load balancer setup—one VM is used as a software load balancer and the other three virtual machines are used for web server farms. It is easily scale-able in real-time traffic and free from the architecture and configuration of virtual machines. The software load balancer is open-source and falls under commercial service as well.
- Virtual Load Balancer: A virtual load balancer acts as a software load balancer, but it is different from software load balancers. The virtual load balancer distributes the web traffic by taking the software program of the hardware load balancer, which was installed on virtual machines.

### 2.2. Load Balancing Measurement Parameter

The main purpose of this section is to give the idea of which parameters we can measure to gauge the performance of the load balancing algorithm. There are always some limitations, restrictions, and some dimension constraints to calculate the load balancing methods, which allow checking whether the given method is worthy of balancing the load or not, which is given as follows:

- Throughput: This parameter imitates the capability of the server. The capability of the server means how much weight it can take. It is one of the important parameters that support calculating the performance of web applications. Maximum throughput is always expected. Throughput is calculated as the number of requests in a given time or transactions per second.
- Average Response Time: It is the aggregate of time used to start satisfying the request of the user after the process of the request.
- Fault tolerance: The capability of the load balancing algorithm that permits the structure to work in some falls down the state of the system.
- Scalability: The algorithm can scale itself according to requisite situations.
- Performance: It is the complete check of the algorithms functioning by seeing precision, price, and quickness.
- Resource utilization: It is used to retain a check on the consumption of the number of resources.

### 2.3. Categorization of Load Balancing Algorithms

Load balancing algorithms can be categorized into two types: static and dynamic algorithms. The static algorithm methodology largely considers the traffic at each instance in the network and distributes the traffic uniformly between all the instances. Dynamic algorithm methodology conversely considers only the current state of the system while making verdicts of allocating the load. In cloud computing, the dynamic algorithm is commonly used for huge networks. round robin and source hashing algorithms are some static algorithms, whereas least-connection, weighted least connection, and least bandwidth algorithms are some examples of dynamic algorithms. The summarized features, advantage, disadvantage and differences of the different load balancing algorithms are presented in the Table 1

#### 2.3.1. Round Robin Algorithm

**Figure 1:**
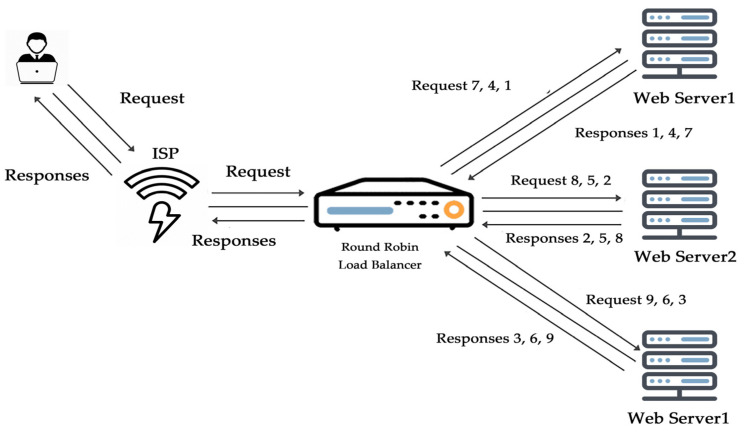
Round robin algorithm.

The round robin algorithm depends on a rotation system or rotationally selects the servers. An incoming request is a substitute to the primary reachable server, and then the server is bounced to the cease of the line, as shown in Figure 1. This algorithm is by and large beneficial while functioning with servers of identical importance. It is the simplest and easy to implement a load balancing algorithm, as it performs best when the resources of the virtual machines are the same.

#### 2.3.2. Weighted Round Robin Algorithm

**Figure 2:**
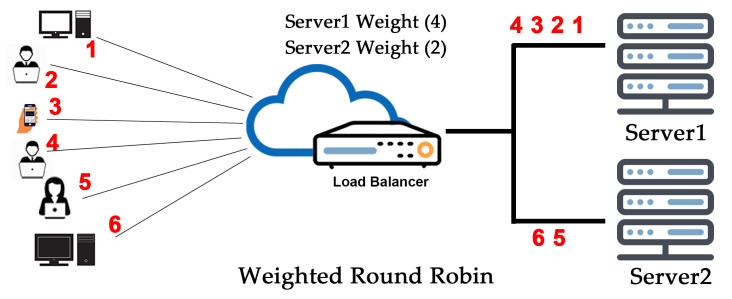
Weighted round robin algorithm.

Each weight server, is used in turn. The weighted round robin algorithm is similar to the round robin one, but is static in nature, so it doesn’t work by changing the weight of the server on the wing. On the other hand, the number of servers is not limited and frequently replicated immediately after a full map is re-published when a server increase. It uses less CPU to run too. A weight is allocated to the respective server established on norms chosen by the cloud system administrator; the most frequently used ways are the server’s visitors‑handling capability. The higher the weight, the higher the percentage of visitor requests the server accepts, as in Figure 2. For example, server1 is allocated a weight of 4 and server 2 a weight of 2. The load balancer forwards four requests to server 1 for each two it sends to server 2. If some virtual machines have more resources in vCPU, Ram, GPU, and other specifications, then the weighted round robin algorithm is used. With the help of weighted round robin, cloud admin can easily distribute the web traffic on high resource virtual machines by defining weight on it. In advance, all the estimation requires implementing this algorithm, and this is a major drawback.

#### 2.3.3. Source Hash

**Figure 3:**
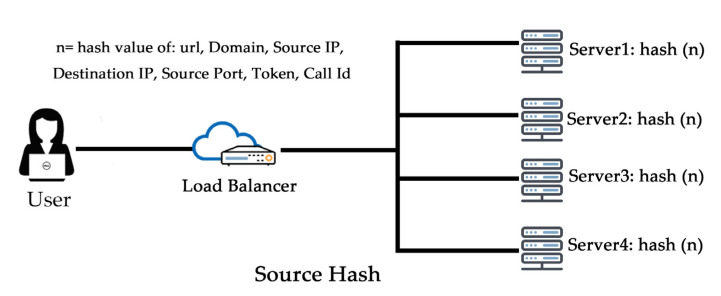
IP hash algorithm.

The source (user’s) IP address absolutely determines which server receives its request on this sincere load balancing algorithm, as in Figure 3. This algorithm is used when the application provider wants the same user route to the same server where the last request has been served. It is very difficult to serve the request of the same server because internet service provider (ISP) provides dynamic IP addresses, so it is difficult to maintain it.

#### 2.3.4. Least Connections

**Figure 4:**
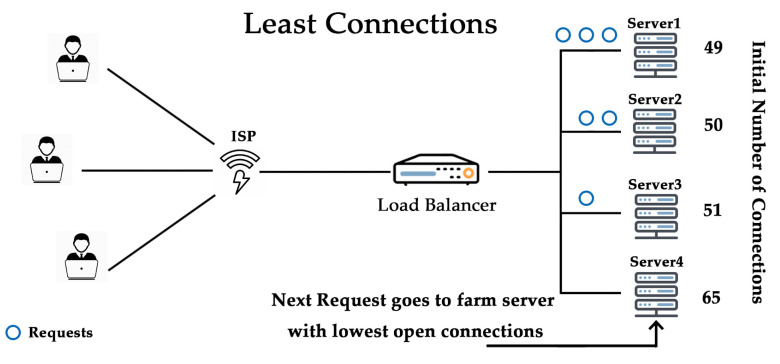
Least connection algorithm.

As its name states, the least connection algorithm instantly sites visitors to whichever server has the least extent of active connections that are supportive throughout heavy visitor’s durations. It facilitates preservation, even distribution amongst all available servers, as shown in Figure 4. Least connections algorithm widely used when longer sessions are required, like MariaDB or SQL, where the transaction rate high, active directory, lightweight directory access protocol (LDAP), etc. This algorithm is not recommended for short sessions applications like HTTP.

#### 2.3.5. Least Response Time

**Figure 5:**
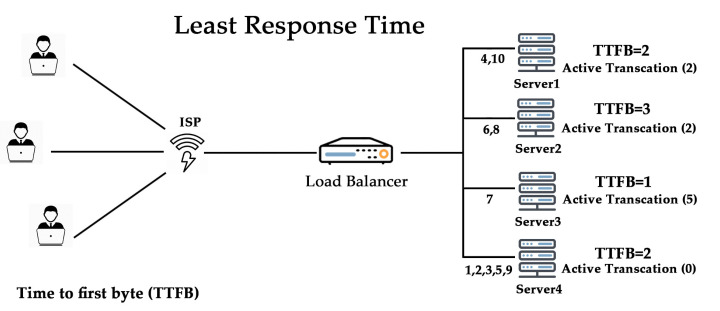
Least response time.

The least response time algorithm directs visitors to the server with the least amount of active connections and the lowest common response time, as shown in Figure 5. Least connections are used where two parameters (least active connections and low response time) are combined with per VM. However, it requires high performing virtual machines; if simple performing virtual machines are used, then the unequal route of traffic might be shown. This algorithm is not recommended for cookie-based session applications.

#### 2.3.6. Least Bandwidth Algorithm

**Figure 6:**
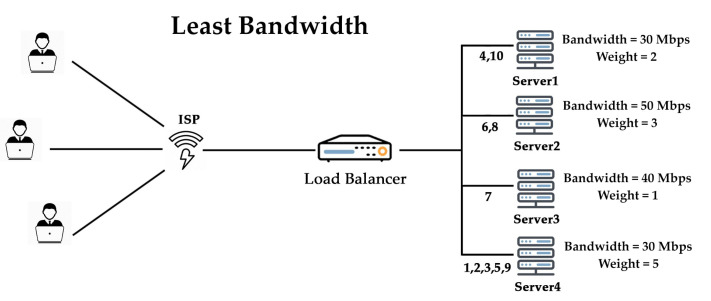
Least bandwidth.

This least bandwidth algorithm processes visitors in megabits (Mbps) per second, sending users’ requests to the server with the smallest Mbps of visitors as given in Figure 6. The least bandwidth algorithm is very handy when all virtual machines in cloud infrastructure have different network bandwidth. It requires approximate network bandwidth, which is difficult to do in networks where the packet size of the data varying, and network bandwidth might be exhausted.

## 3. Related Works

Some people have been done different kinds of works to evaluate the load balancing performance of the many load balancing algorithms in cloud computing. We will reflect on some of this work in this section.

In recent years, result outputs in [6,7,8] have seen incredible growth in communication and computing devices and correlated applications. With this, the necessity for CPU, RAM, bandwidth, storage, and computing power has developed exponentially. In recent times many computing service providers have moved towards the cloud for enhanced scalability and decreased infrastructure price. The work presented incorporates a load balancer at cloud infrastructure with software-defined networking (SDN). The round robin job scheduling is implemented on the load balancer to assign the tasks to the web servers. Time is analyzed to process the requests of the users with and without a load balancer and display the results to help in considering the benefits and drawbacks of using load balancers in the cloud.

Feasibly, the most fascinating part of cloud computing is its flexibility. To be able to use the flexibility of cloud infrastructure, the scalability of infrastructure needs to be scaled horizontally, i.e., it is probably necessary to add and remove assets proposing similar abilities as the existing ones. In such circumstances, a load balancer is commonly used. In order to balance the physical labor, the load balancer automatically able to allocate the load to the recently added servers. Authors show how to renovate the widespread Apache Web Server [9], which is acts as a static load balancer into a cloud-ready dynamic load balancer [10,11].

In cloud load balancing, allocating computing resources is a vital key. Requests should be assigned to avoid the overload of virtual machines and improve the average response time of the audience. It is seen that during hours when request occurrence is high, active VM load balancer (bundled in cloud Analyst) [12] over-allocates initial virtual machines and under-allocates the next ones forming load imbalance. This author suggests a novel [13] VM load balancing algorithm that confirms even the allocation of requests to virtual machines even during hours when requests received in the datacenter are high to ensure quicker response times to the audience. The analysis results propose that the algorithm allocates requests to VM uniformly even during uttermost traffic conditions.

In recent years, cloud computing has led to enormous progress in traffic volume and the number of services demands from cloud servers. This progress leaning of load stances can pose some challenges to the cloud load balancer in the resourceful balancing of the load, which is already an intimidating job. Cloud load balancing is an extremely explored field where plentiful solutions to balance load have been suggested. But no papers provided a wide-ranging review focusing on load balancer as a service model [14]. The authors understand the theories of load balancing, its significance and desired features in the cloud and provide a wide-ranging review of the present load balancing approaches, strengths, and deficiencies. They present a load balancer as a service model adopted by some major market cloud service providers.

The rapid rise in the demand for methodical, commercial, and web applications has led to large scale computation [15]. Cloud computing has arisen as a reasonable, accessible, dependable, scalable, flexible source for web or other applications [16]. To manage web applications requires suitable load balancing and scheduling methods. The authors propose an algorithm based on-time scheduling and priority for load balancing. The method involves the partition of time into various portions and assigning each process to a specific time interval based on priority. The processor attends to the audience request inside the assigned time period. At the end of the time period, the next line up audience request is prepared for implementation. The audience request exits from the line upon accomplishment request; otherwise, the audience waits for its next time period. The rise in waiting time increases the time period the audience requests get in the VM and moderates the overhead of context substituting.

Tasks scheduling algorithms [17] reflect the primary and elementary factors in monitoring cloud computing performance. In this, the authors try to improve scheduling algorithms by emphasizing the load balance to get the best asset consumption, decreasing waiting and executing time performance. They attempt to improve the scheduling algorithm’s performance by suggesting a new approach called “selecting virtual machine with the least load” that can be functional in aggregation with any assignment. Selecting virtual machine with the least load depends on computing the overall load in each VM without deliberation the figure of assignments allotted to it. For measuring the performance accomplished by this technique, it will be applied to conventional simple scheduling algorithms, such as first come first served, shortest job first, and max-min scheduling algorithms.

In cloud load balancing, the static load balancing algorithms share the user request between virtual machines in a cloud datacenter for processing. But there are some problems that arise pertaining to the existing load of each virtual machine. The dynamic algorithms such as “efficient response time load balancer” and “mini time processing load balancer” demonstrate a solution to such problems [18,19]. The importance of these algorithms, before assigning a task, they search the allocation tables on the VM, response time, or the processing time. In this paper, the authors propose a new enhancement of the load balancing by the algorithm “estimated finish time load balancer” [18]. This algorithm takes account of the existing load of the VM of a cloud datacenter and estimates the processing completion time of a task before further any distribution, and try to overcome the glitches caused by static algorithms. The algorithm “estimated finish time load balancer” allows cloud service providers to expand, develop and increase the performance, accessibility and maximize utilization of the use of virtual machines in datacenter.

The author suggested a technique for analyzing the conditions and splits the load balancing methodology into multiple layers. The multi-queue management policy is used to form and split requests in multiple queues depending upon their execution priorities and the rest of the layer handles internal requests of the queue in the cloud network with the help of the network administrator [20]. Due to the handling of both segments, the problem of heavy load processing reduces in many terms like energy utilization, average response time, and network load. All these terms are used to analyze the performance of a VM along with a heavy load on the network. The planned metrics are designed on a .net platform and it attains a total 28% approximate improvement in all the cases.

The cloud environment is a modern development in the IT sector field, which provides computing resources to the users’ on-demand basis. Cloud admin continuously monitors all the computer resources like RAM, vCPU, storage, network, etc., to maintain the load and usage of the resources, so they can get optimal performance of the resources. Ref. [21] did the load balancing in two ways, first non-linear algorithms, and second is scheduling tasks. Non-linear use is for internal load balancing, and second for allocation of computing resources for optimal performance. The experiment will be carried out at the CloudSim [12,22], simulator taking more span because of the parameter for evaluating the outcomes of one of kind algorithm.

Cloud computing is an internet-based methodology where all the IT compute resources are deployed on a cloud with a different datacenter. The HBB load balancing isn’t assigned the task to the right virtual gadget and additionally, it does not recall the quality of service. In order to conquer the disadvantage of the honeybee algorithm, every other algorithm called particle swarm optimization set of rules is used. In the particle swarm optimization set of rules, the venture might be assigned to the virtual system appropriately. IE undertaking will test all of the virtual gadgets and assigns the task to the proper virtual device, which will have minimum memory leak has been considering in quality of services [23]. And the results of the paper suggest methodology can discover superior trade-off answers for assignment scheduling troubles that constitute the possible high-quality compromises the various conflicting objectives.

The author discussed the new hybrid algorithm with the existing load balancing algorithms such as equally spread current execution load, round robin, bee colony optimization, and ant colony optimization [24]. The brand-new hybrid algorithm offers a green load balancing of nodes and performs green scheduling. The hybrid set of rules works on fundamental swarm smart algorithms and ant colony optimization and priority-based bee colony. The above algorithms for scheduling are carried out and cargo balanced within a cloud scenario where the overall performance of load balancing determines which virtual resource is loaded heavily or under loaded. The hybrid algorithm proves to outperform the prevailing processes like Round Robin, Equally Spread Current Execution and ant colony optimization.

It highlights the challenges and features of cloud computing. It discusses the various offerings that cloud computing offers, service layers, virtualization, and load balancing. It introduces the procedures of load balancing static and dynamic, declaring that static load balancing algorithms divide the site visitors equivalently among all servers at the same time as dynamic load balancing is a non-centralized approach. The paper additionally discusses the prevailing load balancing algorithms together with round robin, opportunistic load balancing, randomized algorithm, min-min algorithm, max-min algorithm, active clustering, lock-loose multiprocessing answer for load balancer, ant colony optimization, shortest response time first, based random sampling, active clustering, and honey bee foraging behavior [25].

Reference [26] investigates the different techniques of load balancing algorithms to find the solution for the issues associated with load balancing and venture scheduling in cloud computing. The paper contrasts the static and dynamic load balancing algorithms, pointing out that the static algorithms in overhead phrases are very efficient because they do not need to screen the assets through run-time. Therefore, they might work properly in stable surroundings as their operational residences will not exchange over time and masses are normally regular and unique. On the alternative hand, the dynamic algorithms give higher solutions in order that the properties of the resources at run time can be adjusted dynamically at run-time.

In cloud computing, managing and allocating the responsibilities are the major and most difficult tasks. It requires the ideal deployment of computing resources and also monitoring the overload of the resources. This article uses the ant colony optimization [27] load balancing technique for the route the incoming traffic load dynamically. They describe the two policies first max-min and the second ahead-backward ant tool. The ahead-backward ant tool always checks the neighboring resources for a quick and optimal load transfer process. This paper also shows that pre-planned requirements cannot route the optimal performance of dynamic load balancer for the cloud with much less time. However, it also can also bring network routine below average and severely loaded environments.

Cloud computing has developed one of the most noteworthy services for its power in every part of networking and a new variety of technologies such as security, volume, quality of service, charge, and availability, etc. The major challenge in the cloud network is load balancing. Multiple algorithms were proposed to solve the issues. Some measured fixed variables, while others measured through dynamic variables. In this paper, fixed variables procedures are used with the fresh projected algorithm “MEMA technique” [28]. In the projected algorithm, a few phases are additional to the weighted round robin. Moreover, a comparative study is an analysis between the weighted round robin and MEMA technique is obtainable.

After the evolution of virtualization and cloud infrastructure, cloud computing offers the pay-as-you-go model. It supports cloud computing infrastructure by enlightening the overall performance, enhanced computing assets deployment, power consumption administration, increasing the cloud facilities’ quality of service. In [29,30] the authors study the requirements of assets, design, and web application architecture. They implement the load balancer using general, architectural, and artificial intelligence algorithms according to architecture and requirements. In the end, they evaluate the algorithms and enumerate the positive and negative aspects of the load balancing algorithm.

In this paper, we investigate the load balancer algorithms round robin and least connection algorithm with the help of live cloud infrastructure, which was deployed on the DigitalOcean cloud. We have used open-source services like PHP for a dynamic web application, HAProxy for implementing load balancer algorithms, MariaDB for database, Cloud Firewall for securing the webservers and database server, and Apache Jmeter for testing and analyzing the load on the website [31]. Meanwhile, in the other papers, most authors use the cloud analyst simulation tool to analyze their load balancing algorithms [2,3,4,5]. The Cloud Analyst simulation tool is a pre-defined simulation tool for analyzing algorithms. The use of the cloud analyst simulation tool is now a very important technique for analyzing the load test. We use the quality-of-service parameters to compare the performance and analysis of the algorithms with their overall execution time of each algorithm in milliseconds, average response time, throughput, network bandwidth, and other parameters. The comparison of the works of different authors is summarized in Table 2.

## 4. Methodology

This section shows a comparative study of two of the most commonly used load balancing algorithms. We perform an analysis on the round robin and least connections algorithms. The cloud environment in this paper contains the load balancer server and several target web servers. The virtual users send requests to the load balancer server, which then forwards the request to the target web servers following a standing procedure. The target web servers are supposed to be organized in a parallel topology, which means that once the server finishes processing the requests, it will return the result straight to the virtual user. We used the HAProxy service to implement the algorithms and the JMeter tool for analyzing the results of the algorithms.

In the following subcategories, we define the commonly used cloud simulation tool for introduction purpose only and appropriate comparative research works that apply load balancing algorithms from the cloud sources or cloud audience perceptions.

### 4.1. HAProxy

High Availability Proxy is a free or open-source load balancer, which can load balance very fast, and consistent solutions provide high accessibility, load balancing and a proxy for TCP and HTTP/HTTPS-created web applications. In recent times, with an increase in user traffic, HAProxy is the highly demanding load balancer that easily handles a larger number of audiences. It became the most regular open-source load balancer and distributed with the majority of Linux distributions like RedHat, CentOS, Ubuntu, Fedora, etc. It is also installed by default in cloud virtual machines. Each load balancer server has its own public IP address but shares the same fully qualified domain name. HAProxy [32] backend configuration file is responsible for the load balancing algorithm. HAProxy has nine algorithms, but in this paper, we are testing the two major load balancer algorithms widely used by cloud architecture and cloud service providers.

### 4.2. Round Robin Algorithm

The first and simplest algorithm is Round Robin. We can go on by entering “balance round robin” in the backend section file of HAProxy. With this selection, HAProxy will forward the request over the servers and evenly load your “cluster”. The code example:

backend
  balance roundrobin
  server server1 webserver01:80
  server server2 webserver02:80
  server server3 webserver02:80

In this case, webserver01, webserver02 and webserver03 are the hostname of the servers, we can enter IP addresses also instead of hostname.

### 4.3. Least Connections Algorithm

This load balancer algorithm called “least connections” calculates the number of lively connections for each server. Thus, each following request is distributed to the server with the lowermost number of active connections. We can turn this algorithm on by entering “balance leastconn” in the backend section file of HAProxy. Least Connections is dynamic in nature, which means that server loads may be adjusted on the gentle starts for nodes:

backend
  balance leastconn
  server server1 lcserver01:80
  server server2 lcserver02:80
  server server3 lcserver02:80

In this case, lcserver01, lcserver02, and lcserver03 are the hostnames of the servers; we can enter IP addresses also instead of hostname.

### 4.4. Apache Jmeter

**Figure 7:**
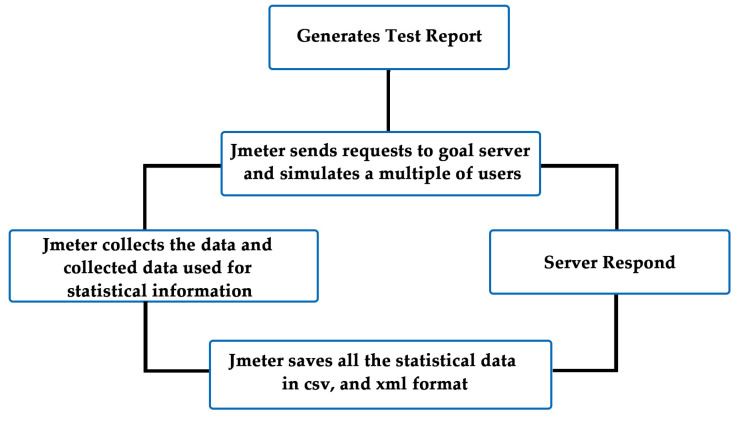
Workflow diagram of Apache Jmeter.

Apache JMeter is a free or open-source, pure Java-based, performance, stress and load testing tool that can be used to analyze the efficient behavior of organizations and calculate the performance of web applications under various load tests. A load test will simulate end-user behavior that methodology the bounds of a web application’s conditions. This tool can be used to design varying or heavy loads on a single or cluster of servers, networks, and concurrent users to test web applications or organizational structure. The work flow chart of the apache jmeter is shown in Figure 7.

Apache JMeter includes various applications, server/protocols:

- Web—HTTP, HTTPS (Java, NodeJS, PHP, ASP.NET)
- SOAP/REST Web services
- FTP
- Database via JDBC
- LDAP
- Message-oriented middleware (MOM) via JMS
- Mail Services like SMTP, POP3 and IMAP
- Native commands or shell scripts
- TCP
- Java Objects

### 4.5. Cloud Analyst Simulation Tool

Cloud Analyst is a java-based framework designed at the University of Melbourne to support the regional distribution of users and datacentres to analyze the cloud infrastructure, resources, and network tools. This simulation tool defines and creates user groups, physical hardware details and generates high traffic for testing the cloud infrastructure. It offers a virtualization technology with complete models for virtual infrastructure to be created and operated in a datacentre. This tool provides an easy to use graphical user interface. The table, diagram and explanation of the potentially high numbers of statistics earned during simulation is given for graphical results. This effective presentation helps identify the respective patterns in performance parameters and facilitates associations between them.

### 4.6. Experimental Setup

In order to implement the suggested round robin and least connections algorithm and compare both algorithms’ effectiveness and performance, the HAProxy load balancer has been configured and utilized. Apache JMeter is used to analyze the results of both algorithms. This section provides information about the cloud service provider, virtual machines, resources, cost and other software used for load testing setup. It also explains the design of the HAProxy load balancer and Apache JMeter and the necessary modules involved, followed by the implementation of the least connection algorithm and round robin algorithm.

#### 4.6.1. Virtual Machines Setup and Software

DigitalOcean Inc. [33] is an American cloud infrastructure company. Its headquarters are situated in New York City and datacenters are present worldwide. DigitalOcean offers creators cloud facilities that help set up and scale applications that run concurrently on several workstations. Since January 2018, DigitalOcean was the third-largest cloud hosting provider in the world in positions of web-facing workstations.

We designed simple cloud architecture for the comparative and analysis of different load balancer algorithms. we used a total of ten virtual machines for the cloud architecture implemented in DigitalOcean. Five virtual machines are used for the RR algorithm and the remaining five virtual machines are used for LC algorithm, as shown in Table 3 The following are the configuration of virtual machines, which was used in the demonstration.

#### 4.6.2. Primary Setup of Virtual Machines

The important sections and services are set up in all virtual machines to use for demonstration and each service is explained in detail below.

##### Datacentre Regions

This section is used to specify the terrestrial sites of the cloud architecture where the resources are allotted. In general, DigitalOcean provides eight datacenter regions of different continents in total. The datacenter provides the infrastructure to the cloud architecture design by audience/users or cloud design. Each datacenter consists of a number of virtual machines. All the resources: Ram, storage, IP Address, firewall, core, etc. and virtual machines have their own specifications.

##### Apache Server

The Apache HTTP Server or Apache is an open-source web server software program, launched under Apache License 2.0. Apache has been evolved and maintained with the aid of public builders under the sponsorships of the Apache software program foundation [34]. The large majority of Apache net Server nodes run on a Linux operating system; however, present variations also run on Microsoft home windows Server and a varied variety of Unix-like operating systems. Apache permits customers to serve content like textual content, photographs, audio and video, and so forth on the internet- therefore the name “webserver”.

##### PHP

PHP Hypertext Pre-processor is a server-side programming language and a dominant program for designing the making of dynamic and interactive web applications and pages. PHP is the commonly used, free, and well-organized scripting language and it is an alternative to challengers such as Microsoft’s ASP. PHP 7 is the newest stable release.

##### MariaDB

MariaDB is a community-advanced, commercially supported department of the MySQL Relational Database Management System (RDBMS), expected to stay unfastened and open-source software beneath the GNU General Public License. Improvement is led by a number of the unique programmers of MySQL who left it because of concerns over its getting hold of by way of Oracle organization in 2009.

##### Cloud Firewall

Firewalls place a barricade between servers and other digital devices or other devices on the network to guard them against exterior malicious traffic like viruses, DDOS attacks, and hackers. Firewalls can be host-based, which are constructed on a per-server source using daemons like IPTables or UFW. Cloud firewalls are network-based and halt traffic at the network layer before it reaches the server.

#### 4.6.3. Implementation of the Round Robin Algorithm

**Figure 8:**
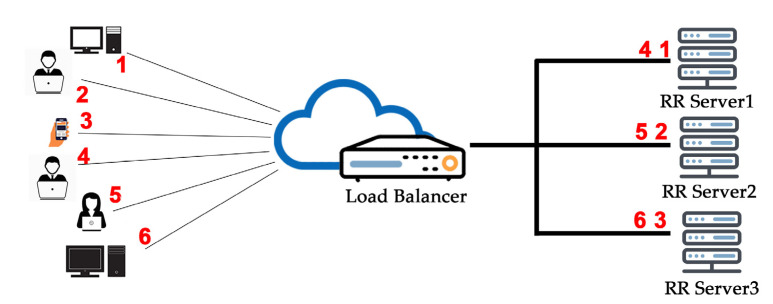
Model of the round robin algorithm.

In this subsection, we described step by step implementation of the round robin algorithm, and the figure described the basic model of round robin how the requests are served in the backend shown in Figure 8. The implementation steps of Round Robin Algorithm is given below.

1. The first request of users comes to HAPrxoy Load Balancer, as shown in Figure 8.
2. HAProxy Load Balancer selects which VM should get incoming requests.
3. The first request of the user is assigned to any random VM.
4. Once the first request is assigned, virtual machines are ordered in a cyclic manner.
5. Virtual machine which received the first user request is moved back to all virtual machines.
6. The next request of users is assigned to the next VM in cyclic order.
7. Go to Step 3 for each user request until Load Balancer processes all requests.

#### 4.6.4. Implementation of the Least Connections Algorithm

**Figure 9:**
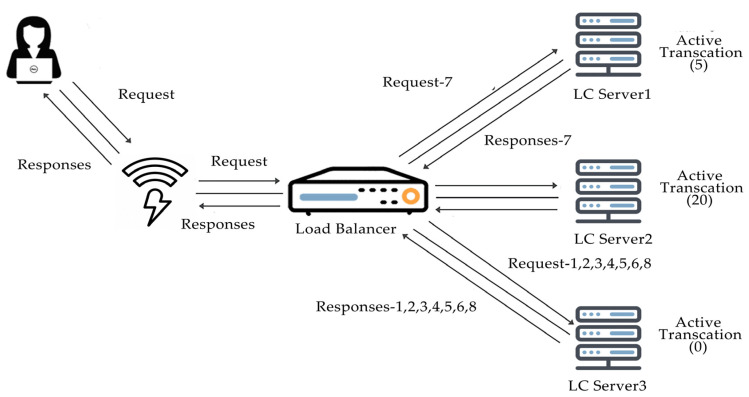
Model of the least connections algorithm.

This subsection described step by step implementation of the least connections algorithm shown below. The figure described the basic model of least connections how the requests are served by the least connections in the backend shown in Figure 9. The implementation steps of the Least Connections Algorithm is given below.

(1) The first request of users comes to HAPrxoy Load Balancer, as shown in Figure 8.
(2) HAProxy Load Balancer selects which VM should get incoming requests.
(3) The first request of the user is assigned to any random virtual machine.
(4) Once the first request is assigned, virtual machines are ordered in the least amount of connections that have the least amount of requests, then assign the new request to that VM.
(5) VM, which received the first user request, is moved back to all virtual machines after completing all the user requests.
(6) The next request of users is assigned to the next VM in the least number of connections.
(7) Go to Step 3 for each user’s request until load balancer processes all requests.

**Figure 10:**
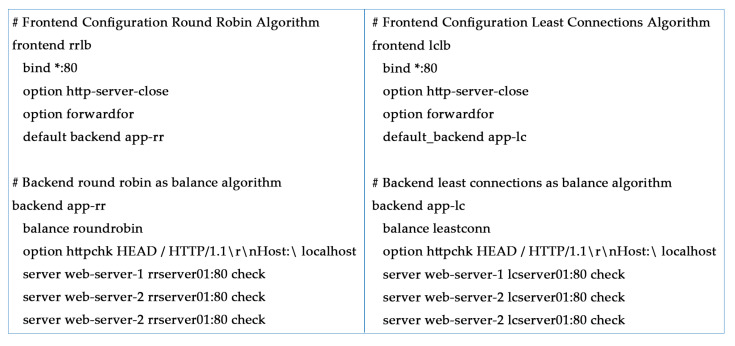
The configuration code of the RR and LC algorithms.

How to configure the round robin and least connections in the algorithm in the HAProxy configuration file are mentioned in Figure 10.

## 5. Results & Evaluation

This section features a detailed discussion about the setup of cases for performing load testing on load balancer servers and explains the results of each case. The quality of service parameters used to compare the performance and analysis of the suggested algorithms with their overall execution time of each algorithm in milliseconds, response time, average execution time, 90% percentile, 95% percentile, 99th percentile throughput, network bandwidth, active threads over time, latency over time and more.

**Figure 11:**
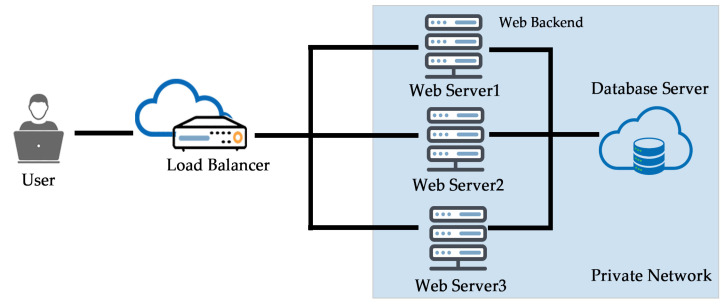
Cloud architecture implementation on DigitalOcean.

In this task, we set up cloud architecture on DigitalOcean and host one dynamic website to test RR and LC algorithms, as shown in Figure 11. This experimental result focuses on different cases that were designed for scheduling incoming requests.

### 5.1. First Load Test

**Figure 12:**
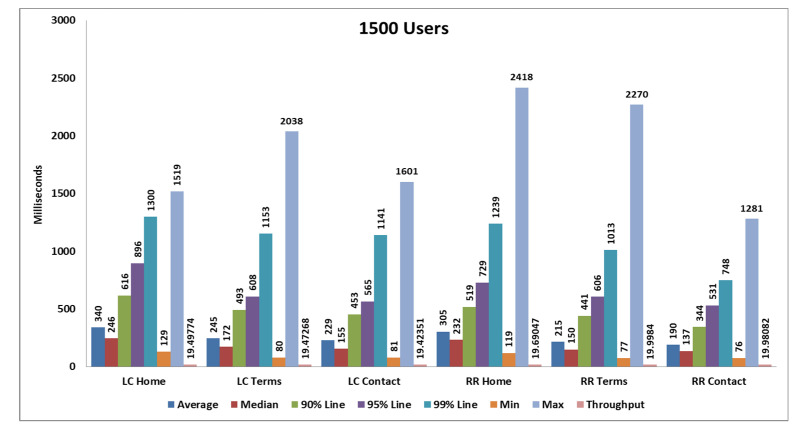
Aggregate graph using Table 3 data that shows average response time, median, 90%, 95%, 99%, min, max, and throughput parameter of combined graph of the RR and LC algorithms.

**Figure 13:**
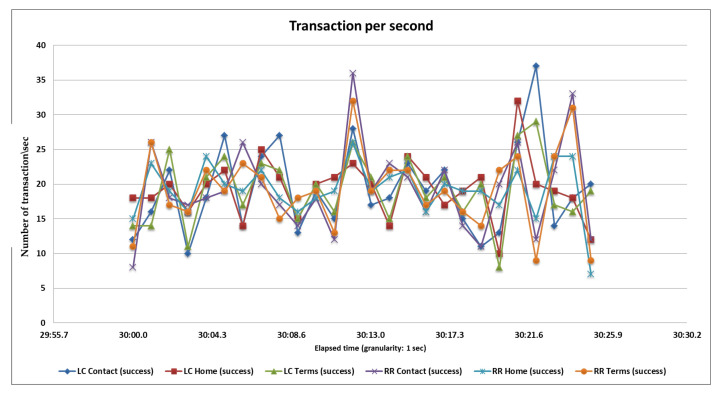
Throughput or transactions per second of the RR and LC algorithms.

We perform our test with 1500 samples (samples means users), 1500 samples are divided into three pages and each page serves 500 samplers and every 20 s, 25 samples are hitting the server. After completing all samplers’ requests, we generate tabular reports as well as graphs. Performance analysis with different parameters on two algorithms and their statistical results and graphs obtained from the first load test are shown in Table 4 and in Figure 12 and Figure 13.

### 5.2. Second Load Test

**Figure 14:**
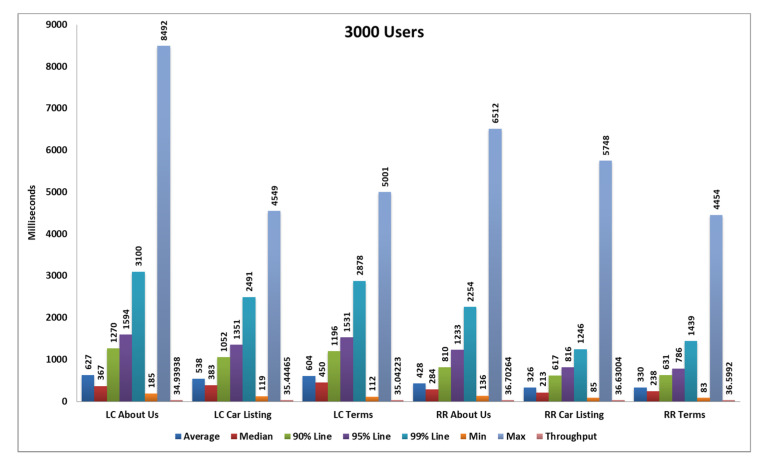
Aggregate graph using Table 4 data, showing the average response time, median, 90%, 95%, 99%, min, max, and throughput parameter of combined graph of the RR and LC algorithms.

**Figure 15:**
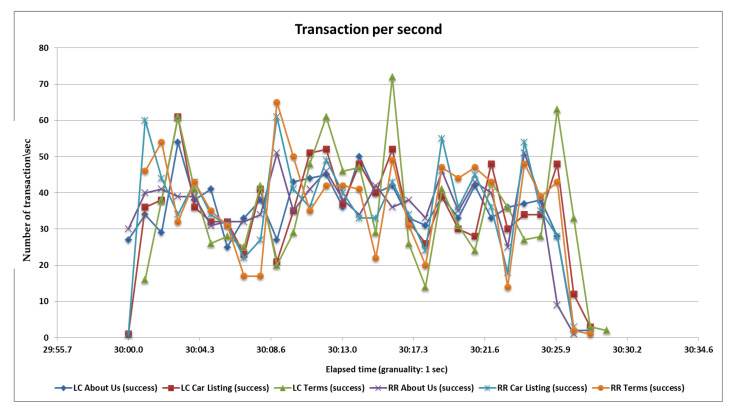
Throughput or transactions per second of the RR and LC algorithms.

In the second case, we perform a test with 3000 samples, divided into three pages and each page serves 1000 samplers and every 40 s, 25 samples are hitting the server. After completing all samplers’ requests, we generate tabular reports as well as graphs. Performance analysis with different parameters on the two algorithms and their statistical results and graphs obtained from the second load test are shown in Table 5, Figure 14 and Figure 15 below.

### 5.3. Third Load Test

**Figure 16:**
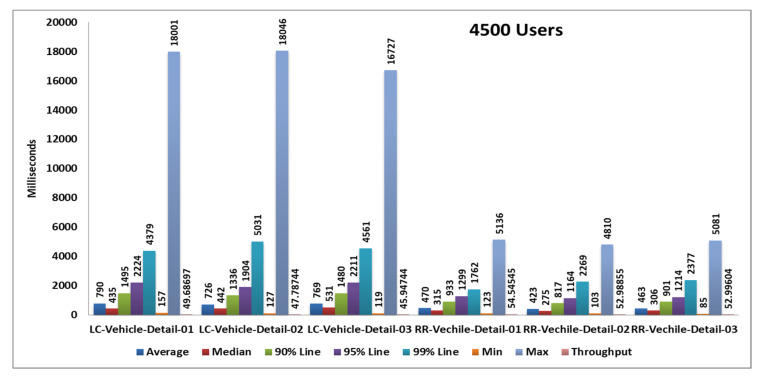
Aggregate graph using Table 5 data. It shows the average response time, median, 90%, 95%, 99%, min & max parameters of the combined graph of the RR and LC algorithms of Table 4.

**Figure 17:**
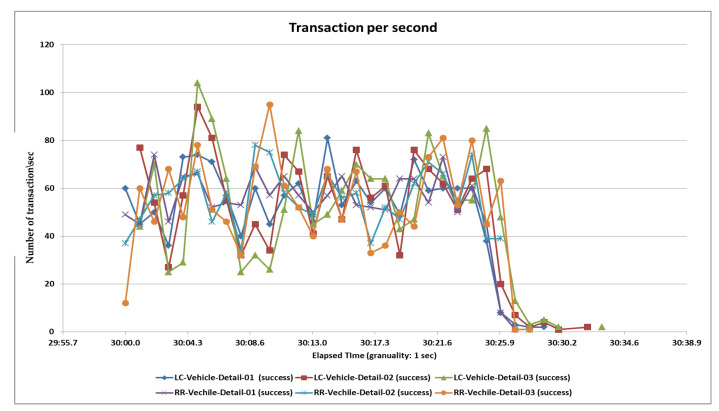
Throughput or transaction per second of the RR and LC algorithms.

In the third case, we test 4500 samples divided into three pages and each page serves 1500 samplers and all samplers’ hitting the server in the span time of 1 min. After completing all samplers’ requests, we generate tabular reports as well as graphs. Performance analysis with different parameters on two algorithms and their statistical results and graphs obtained from the second load test are shown in Table 6, Figure 16 and Figure 17.

### 5.4. Fourth Load Test

**Figure 18:**
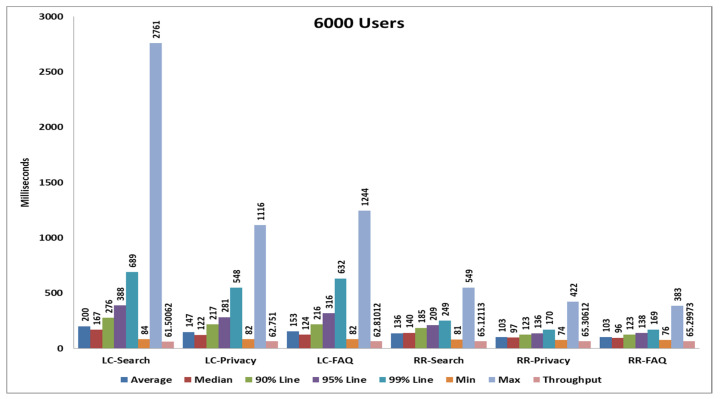
Aggregate graph using Table 6 data, showing the average response time, median, 90%, 95%, 99%, min & max parameter of the combined graphs of the RR and LC algorithms of Table 5.

**Figure 19:**
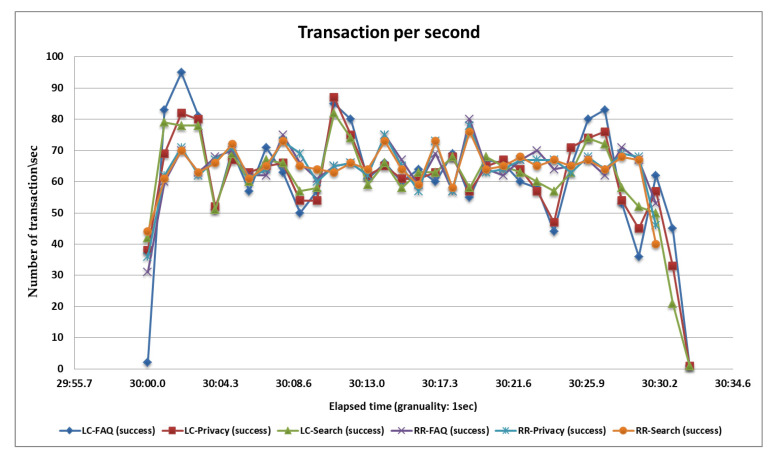
Throughput or transactions per second of the RR and LC algorithms.

In the fourth case, we perform the test with 6000 samples divided into three pages and each page serves 2000 samplers and all samplers’ hitting the server at the same time. After completing all samplers’ requests, we generate tabular reports as well as graphs. Performance analysis with different parameters on the wo algorithms and the statistical results and graphs obtained from the second load test are shown in Table 7, Figure 18 and Figure 19.

After performing all the sample load tests, we found that the average response time, throughput time, received KB/s, Sent KB/s results show that the round robin algorithm performs better than the least connections algorithm. Table 2, Table 3, Table 4 and Table 5 show that the average response times of the round robin algorithm are 236, 361, 452 and 114 milliseconds, respectively. In contrast, the least connections times are 271, 590, 762 and 167 milliseconds, respectively. When comparing average response time, we have thus found that the round robin algorithm takes less time to serve all sample requests. For the second parameter, the throughput times of the round robin algorithm are 58.53, 106.37, 156.21 and 194.14 milliseconds, respectively, and for the least connection algorithm they are 57.03, 102.06, 132.13 and 182.92 milliseconds, so in the throughput results, the round robin algorithm also performs better because a higher throughput value is always better. The third parameter is network bandwidth, and it includes received KB/s, and sent KB/s. The round robin received KB/s values are 1371.83, 2469.61, 4006.99, 3954.47, and the sent KB/s values are 7.37, 13.85, 21.66 and 25.53 KB/s, while for the least connections algorithm, the receive KB/s values are 1336.37, 2369.54, 3389.18 and 3726 KB/s, and the sent KB/s values are 7.02, 12.99, 17.94 and 23.52 KB/s. This result also shows that the round robin algorithm network bandwidth utilization also performs better as compared to the least connections algorithm. We have also compared the statistics tables and graphs of the different cases, and the overall average response time of the round robin algorithm is low, and the throughput is high as compared to the least connections algorithm. The network bandwidth is also high in the the round robin algorithm. Therefore, the round robin algorithm performs better than the least connections algorithm. The summarized result analysis is shown in Table 8.

## 6. Conclusions

Cloud computing is widely used in industry. However, there are numerous standing issues like server migration, load balancing, power management, network management, virtual machine relocation, etc. In a cloud infrastructure, load balancing is a major task and a key challenge to distribute the incoming or dynamic traffic workload proficiently and rightfully to all the cloud datacenters to achieve audience satisfaction and optimal resource usage apportion. It is a huge challenge to choose an algorithm that reduces the overall average response time and it should be cost-effective too. In this paper, a comparison of the two round robin and least connections load balancing algorithms helps choose the best load balancing algorithm for any particular situation. The statistical results of the paper showed that if the average response time of the algorithm is low and throughput is high, that means the algorithm performs better on the provided cloud infrastructure. This research work can promote a superior algorithm by comparing and adding the different parameters like sticky sessions, redirection rules, cookie-based sessions, etc. As future work, we will extend the current work in multiple ways, like comparing existing load balancing algorithms such as weighted round robin, source hash, least response time, and least bandwidth. We can also use the load balancing algorithms on the database server where database transactions are high and compare the performance of the database server with or without a load balancing algorithm. In this manner, the objective is to balance the audience traffic of the cloud infrastructure while enhancing the execution, reducing the overall response time, increasing the throughput for a particular number of jobs, and proficiently handle resource usage.

## Figures and Tables

**Table 1:** The summarized features, advantage, disadvantage and differences of the different load balancing algorithms.

Algorithm	Request Serve Method	Nature	Advantage	Disadvantage
Round Robin	The request is given to the server in a rotated Sequential Manner	Dynamic	Easy to configure, deploy and widely used algorithm	Servers can overload and crash if they have different resources and processing capacities.
Least Connections	The request is given to the server with the lowest number of connections.	Dynamic	Avoids overloading a server by verifying the number of server connections	When calculating the number of existing connections, the server capacity cannot be considered.
Weighted Round Robin	Every server is used, in turn, by weight.	Static	Can send more requests to more capable and loaded servers	All the estimation requires implementing this algorithm, and this is a major drawback and also requires estimating IP networks with different packet sizes, which are difficult to do.
Source Hash	The source IP address shall be hashed and divided by the total number of servers operating to decide which server the request receives.	Static	Users connect to a still active session after disconnection and reconnection. It will increase performance.	Internet Service Provider (ISP) provides dynamic IP addresses, so it is difficult to maintain them.
Least Response Time	The request is given to the server with the lowest response time.	Dynamic	Consider both the server’s capacity, response time and the number of current connections to avoid overload and crash.	Simple performing virtual machines are used, then the unequal route of traffic might be shown and this algorithm is not recommended for cookie-based session applications.
Least Bandwidth	Every server is used, in turn, by network bandwidth.	Static	Can send more requests to more capable and network bandwidth loaded servers.	It requires approximate network bandwidth, which is difficult to do in networks where the packet size of the data varying, and network bandwidth might be exhausted.

**Table 2:** Comparison of Related Works.

Author (Year) [Reference]	Objective	Method/Technique/Tool	Analysis
Manaseer (2019) [29]	Reduce response time for vital request	Fixed variables algorithm “MEMA Technique”	Adding few steps in weighted round robin (wrr) improves the distribution of traffic through servers
Tahani Aladwani. (2017) [17]	Scheduling algorithms for monitoring and improve cloud computing performance.	Selecting Virtual Machine with the least load	Get the best assets consumption, decreasing waiting and executing time performance.
M. Al-Ayyoub (2016) [20]	Analyses the conditions and splits the load balancing methodology into multiple layers.	Multi-agent framework	Reduces energy utilization, average response time, and network load. An approximate 28% improvement showed.
Ren Gao (2015) [28]	Ahead-backward a tool to find nearest resources for a quick and optimal load transfer.	Ant Colony Optimization	Uses Ant Colony Optimization to route the incoming traffic load dynamically.
M. Rahman (2014) [14]	Focus on load balancer as a business model and importance in a cloud environment	Load Balancer as a Service Model	Present load balancer as a service model, and adopt the best service for optimal performance.
Y. Fahim (2014) [18]	Try to overcome the glitches caused by static algorithms.	Estimated finish time load balancer	Increase the performance, accessibility and maximize utilization of the use of virtual machines
H.C. Hsiao (2013) [27]	Reduce the dynamic load imbalance in a distributed file system in a cloud environment	MapReduce programming paradigm	MapReduce is performed in parallel over the servers and improve the performance and reduce the imbalance of load.

**Table 3:** Configuration of virtual machines used in cloud architecture for analysis of load balancer Algorithm.

S. No.	Name	vCPU	Memory	Storage	Qty	Cost
1	Web Servers for Round Robin	1 Core	2 GB	50 GB SSD	3	$10/month
2	Database Server for Round Robin	1 Core	1 GB	25 GB SSD	1	$5/month
3	Web Servers for Least Connections	1 Core	2 GB	50 GB SSD	3	$10/month
4	Database Server for Least Connections	1 Core	1 GB	25 GB SSD	1	$5/month
5	Load Balancer Virtual Machine for Round Robin	1 Core	2 GB	50GB SSD	1	$10/month
6	Load Balancer Virtual Machine for Least Connections	1 Core	2 GB	50GB SSD	1	$10/month

**Table 4:** Statistics table of the RR algorithm and LC algorithm for the first load test.

Label	# Samples	Average	Median	90% Line	95% Line	99% Line	Min	Max	Error %	Throughput	Received KB/s	Sent KB/s
**RR** **Home**	500	305	232	519	729	1239	119	2418	0.00%	19.69047	499.93	2.38
**RR** **Terms**	500	215	150	441	606	1013	77	2270	0.00%	19.9984	466.76	2.62
**RR** **Contact**	500	190	137	344	531	748	76	1281	0.00%	19.98082	431.23	2.52
**TOTAL**	1500	236	181	438	615	1191	76	2418	0.00%	58.53202	1371.83	7.37
**LC** **Home**	500	340	246	616	896	1300	129	1519	0.00%	19.49774	495.04	2.3
**LC** **Terms**	500	245	172	493	608	1153	80	2038	0.00%	19.47268	454.49	2.49
**LC** **Contact**	500	229	155	453	565	1141	81	1601	0.00%	19.42351	419.2	2.39
**TOTAL**	1500	271	208	525	672	1255	80	2038	0.00%	57.03205	1336.67	7.02

**Table 5:** Statistics table of the round robin algorithm and least connection algorithm of the second load test.

Label	# Samples	Average	Median	90% Line	95% Line	99% Line	Min	Max	Error %	Throughput	Received KB/s	Sent KB/s
**RR About Us**	1000	428	284	810	1233	2254	136	6512	0.00%	36.70264	727.17	4.87
**RR Car Listing**	1000	326	213	617	816	1246	85	5748	0.00%	36.63004	970.62	4.65
**RR Terms**	1000	330	238	631	786	1439	83	4454	0.00%	36.5992	854.22	4.79
**TOTAL**	3000	361	245	668	987	1594	83	6512	0.00%	106.37166	2469.61	13.85
**LC About Us**	1000	627	367	1270	1594	3100	185	8492	0.00%	34.93938	692.24	4.54
**LC Car Listing**	1000	538	383	1052	1351	2491	119	4549	0.00%	35.44465	939.21	4.4
**LC Terms**	1000	604	450	1196	1531	2878	112	5001	0.00%	35.04223	817.88	4.48
**TOTAL**	3000	590	387	1212	1520	2833	112	8492	0.00%	102.06165	2369.54	12.99

**Table 6:** Statistics table of the round robin algorithm and least connection algorithm of the third load test.

Label	# Samples	Average	Median	90% Line	95% Line	99% Line	Min	Max	Error %	Throughput	Received KB/s	Sent KB/s
**RR-Vechile-Detail-01**	1500	470	315	933	1299	1762	123	5136	0.00%	54.54545	1391.02	7.56
**RR-Vechile-Detail-02**	1500	423	275	817	1164	2269	103	4810	0.00%	52.98855	1391.05	7.35
**RR-Vechile-Detail-03**	1500	463	306	901	1214	2377	85	5081	0.00%	52.99604	1335.3	7.35
**TOTAL**	4500	452	309	884	1246	2246	85	5136	0.00%	156.2175	4006.99	21.66
**LC-Vehicle-Detail-01**	1500	790	435	1495	2224	4379	157	18001	0.00%	49.68697	1267.11	6.74
**LC-Vehicle-Detail-02**	1500	726	442	1336	1904	5031	127	18046	0.00%	47.78744	1254.51	6.49
**LC-Vehicle-Detail-03**	1500	769	531	1480	2211	4561	119	16727	0.00%	45.94744	1157.7	6.24
**TOTAL**	4500	762	475	1439	2096	4569	119	18046	0.00%	132.1314	3389.18	17.94

**Table 7:** Statistics table of the round robin and least connections algorithms for the fourth load test.

Label	# Samples	Average	Median	90% Line	95% Line	99% Line	Min	Max	Error %	Throughput	Received KB/s	Sent KB/s
**RR-Search**	2000	136	140	185	209	249	81	549	0.00%	65.12113	1446.27	8.59
**RR-Privacy**	2000	103	97	123	136	170	74	422	0.00%	65.30612	1295.92	8.67
**RR-FAQ**	2000	103	96	123	138	169	76	383	0.00%	65.29973	1244.07	8.48
**TOTAL**	6000	114	100	154	175	233	74	549	0.00%	194.1496	3954.47	25.53
**LC-Search**	2000	200	167	276	388	689	84	2761	0.00%	61.50062	1365.87	7.93
**LC-Privacy**	2000	147	122	217	281	548	82	1116	0.00%	62.751	1245.22	8.15
**LC-FAQ**	2000	153	124	216	316	632	82	1244	0.00%	62.81012	1196.64	7.97
**TOTAL**	6000	167	136	255	336	618	82	2761	0.00%	182.9324	3726	23.52

**Table 8:** The summarized comparison result of the round robin and least connections algorithms.

	Average Response Time (in milliseconds)	Throughput (in milliseconds)	Received (KB/s)	Sent (KB/s)
Round Robin	290.75	128.815	2950.73	17.10
Least Connections	447.5	118.54	2705.35	15.37
Desired Value	Lower Value (290.75) is better	Higher Value (128.815) is better	Higher Value (2950.73) is better	Higher Value (17.10) is better
Result	Round Robin	Round Robin	Round Robin	Round Robin
